# Use of waste fish oil biodiesel blended with aluminium oxide nanoparticle in IC engines: an experimental on performance, combustion and emission study

**DOI:** 10.1038/s41598-022-17059-5

**Published:** 2022-07-28

**Authors:** B. Thamarai Kannan, T. Sathish, Ravishankar Sathyamurthy, Kuma Gowwomsa Erko

**Affiliations:** 1 Department of Mechanical Engineering, KPR Institute of Engineering and Technology, Arasur, Coimbatore, 641407 Tamil Nadu India; 2 Department of Mechanical Engineering, Saveetha School of Engineering, SIMATS, Chennai, 602 105 Tamil Nadu India; 3 Department of Mechanical Engineering, King Fahd University of Petroleum and Minerals, Dammam, Dhahran, Saudi Arabia; 4 Department of Mechanical Engineering, University Centre for Research & Development, Chandigarh University, Gharuan, Mohali, Punjab India; 5 Department of Mechanical Engineering, Ambo University, Ambo, Ethiopia

**Keywords:** Energy science and technology, Engineering

## Abstract

Alternate fuels are in great need as the world's natural resources are depleting with continuous consumption. Furthermore, with a continuous increase in the use of conventional fuel which emits a large number of harmful pollutants to the environment and thus increasing global warming, the need for alternative fuel is in great need. This investigation thus focused on the impact identification on the use of biodiesel from fish waste-based biodiesel [BDWFO (Bio-Diesel of Waste Fish Oil)] with Nanoparticles in single cylinder water cooled IC engine. The fish wastes in fish processing industries/fish markets are used to produce oil and its biodiesel is produced by the transesterification method. The individual BDWFO, Diesel, and blends of 20% of BDWFO were tested with the engine. Then another two combinations of fuel created 200 ppm of 40 nm Aluminium Oxide nanoparticles (AN) mixed with BDWFO, blends of 20% of BDWFO. These five fuels were considered to study the engine performance, combustion, and emissions from the exhaust. The experimental results confirmed the presence of aluminium oxide nanoparticles in BDWFO provides improved engine performance and reduced emissions from exhaust gas except for CO_2_.

## Introduction

With increasing demands in mobility and industrialization, there is a constant urge for Fossil fuels for IC engines as they are the prime source of energy for Automobiles and industrial power generators. The usage of fossil fuels, on the other hand, results in a significant quantity of greenhouse gases being released into the atmosphere, which will have a negative impact on the climate. Also, the increasing demands for fossil fuels have depleted the crude oil reserves. In order to restrain the usage of IC engines in near future, the engines have to run using other cleaner and alternative fuels. So, the need for the identification of alternate fuels and their properties enhancement should be explored.

As a potential replacement in internal combustion engines, biodiesel has constantly attracted attention due to its comparable characteristics to diesel and its ability to burn with reduced exhaust emissions. Ramadhas et al.^1^ studied the effect of vegetable oils as alternative fuels for diesel engine applications. They mentioned the different production methods of biodiesel like transesterification, thermal cracking and etc., desired properties like CN, viscosity, flash and fire point, Calorific values and etc., and their properties improvisation techniques.

The impact of higher alcohol content in biodiesel on engine performance and emission parameters was experimentally analyzed by Yilmaz et al.^2^. Propanol, butanol, and pentanol with 10% concentration were blended with biodiesel and it was reported that the use of higher alcohol content in the biodiesel reduces the viscosity, heating value, and cetane index of neat biodiesel. Results showed that the use of pentanol with biodiesel significantly reduced the BSFC while compared to propanol and butanol in addition to neat biodiesel. The decrease in the BSFC using pentanol as an additive with biodiesel may be due to higher heating value and lower latent heat for vaporization. With higher BSFC and higher BTE, the EGT emitted from the engine using 10% pentanol from the fuel after combustion increases^2^.

Syed Ameer Basha et al.^3^ also discussed the complete details of alternate fuels from vegetable oils, animal fats, and synthetic elements. So many vegetable oils can be still studied^4–10^ based on increasing the engine efficiency, combustion performance and reducing the emission from the engine exhaust when using those vegetable oils such as Neem oil^4^ and^5^, Pongamia oil^6^, Cotton seed oil^7^ and^8^, Caster oil^9^, Camphor oil^10^, Cashew nut oil^10–12^. Bayonle Kayode et al.^13^ evidently used the transesterification techniques in different categories to produce the bio-diesel with a clear structure of the arrangements. They listed the various catalysts for the transesterification process method. But these all are only focused on vegetable oils, not animal fatty acids.

Yahyaee et al.^14^ give the complete details about the oil extraction from the fish wastes and they recommended that can be used as biodiesel in engines. This production method is only based on the fish wastes in the fish market. They give the idea about the recycling or reuse of the wastes of the fishes. Every day tones and tones of the fish wastes were available in the fish markets which can be used as a useful products such as biodiesel. They use a separate machine to extract the oil from the wastes of fish (like head, wings, skin, bone and etc.,) and they followed the ASTM standard. They have used the transesterification method with methanol and KOH to produce biodiesel to obtain the maximum yield.

Ayatallah Gharehghani et al.^15^ investigated the biodiesel from waste fish oil utilization on the compressed ignition engine. They studied the performance, combustion parameters, and emission norms with an analysis of energy balancing in a clear manner in the variable compression ratio engine (Ricardo E6—Single cylinder). They compare those things with the fuel of Diesel and Waste fish oil biodiesel (1005) and its blends of 25%, 50%, and 75% on a volume basis. They reported that the combustion losses takes place by nearly 15.2%, efficiency increased by 2 to 5% for its blends, 5.2% of CO emission, and 11.6% of HC emission was nearly reduced by the blends when compared with the diesel fuel.

Godiganur et al.^16^ investigated the fish oil biodiesel-based engine operating characteristics and mentioned that the fish oil methyl ester used in diesel engines were consume more fuel when compared with the normal diesel used in the same engine. It will lead to the consumption of the fuel used in the engine. Sahil Gupta et al.^17^ investigated and compared the blends of fish oil (20%) and blends of neat Mahua oil a single-cylinder direct injection diesel engine with the power of 5.9 kW at 1500 rpm of maximum speed, a 17.5 compression ratio. Fish oil blends produce higher Break thermal efficiency, Carbon monoxide, and Carbon dioxide emissions than blends of Mahua oil. Blends of these two oils reduce the smoke opacity when compared with diesel fuel. They showed that the oxides of nitrogen also get increased when compared with traditional fuel.

Srikanth^18^ investigated the diesel engine with a single cylinder using diesel, biodiesel from fish oil, and blends of ethanol with biodiesel and diesel, and a comparison was made to assess the engine performance and emission characteristics. Blends of biofuel with ethanol lead to higher efficiency than the other two fuels. Both biodiesels lead to higher consumption of fuel and exhaust gas temperature was lowered while compared with diesel. Similarly, the CO emission decreased and the HC emission increased while the NOx emission increased with loads. Presence of the ethanol leads to a reduction in the smoke opacity when compared with the other fuels.

Sakthivel et al.^19^ derived the fish oil from the fish processing industries as the industry particularly focused on the main product, while the by-product from the industry was used for processing the waste fish oil. The wastes of those fish parts can be used to produce the waste fish oil which leads to meet the energy crisis of the conversion fuels such as petroleum products and coal-based fuels. It was concluded that the fish oil obtained from the process has the Cetane number of 52.6 and a calorific value of 40,050 kJ/kg. This fish oil leads to higher combustion, and performance with a considerable reduction in the emission from the compressed ignition engine when compared with diesel fuel. It was also concluded that adding cetane number improvers can cause considerable alteration in the combustion process. Atmanli^20^ investigated the effects of 2-ethylhexyl nitrate (EHN) as a cetane improver on engine characteristics. EHN at 500, 1000, and 2000 ppm were added to microemulsions of various compositions and a detailed study on engine characteristics of Turbocharged DI diesel was studied. The studies showed that there is a reduction in BSFC and oxides of nitrogen while the study revealed that there is an increase in Hydro carbon and CO emissions.

The assessment of engine characteristics using waste oil biodiesel with n-pentanol at different proportions was examined by Yilmaz and Atmanli^21^. The pentanol concentration varied from 5 to 20%, while the diesel fuel proportion varied from 60 to 80%. The waste oil biodiesel in diesel and alcohol blends was limited to 20%. However, the addition of alcohol content in the biodiesel and diesel blend decreased the kinematic viscosity which simultaneously decreased the cetane index of the fuel. Results depicted that the influence of higher pentanol content in the blend increased the exhaust gas temperature and BSFC while the engine operated in full load condition. With higher latent heat of vaporization of pentanol, the HC and CO emissions increased while the optimum pentanol share in the fuel is found to be 5%. Due to higher oxygen available in the fuel, the formation of NOx significantly increased which may also be attributed due to higher cylinder temperature during the combustion.

Atmanli^22^ analyzed the effect of engine performance and emission characteristics by using the ternary blends of diesel, biodiesel, and higher alcohols such as propanol, n-butanol, and pentanol. The addition of higher alcohol managed to reduce the combustion temperature and NOx emission with the compromise in thermal efficiency.

Yilmaz and Davis^23^ carried out experiments on a CI diesel engine fuelled using higher concentrations of n-butanol in waste oil biodiesel and diesel blends to study the effect of Polycyclic aromatic hydrocarbons which appeared to be an unregulated emission. Results showed that the fuel properties such as density, kinematic viscosity, lower heating value, and cetane index significantly reduced the increased concentration of n-butanol. At lower engine load, the BSFC increased with an increase in the blend of n-butanol while at higher engine load, the BSFC increased as the engine was operated at increased speed. The engine speed significantly plays a role in the amount and time of fuel to burn. The increased BSFC at the higher blend of n-butanol in biodiesel may be due to the lower cetane index and high enthalpy of vaporization. The higher flame propagation by the n-butanol with 40% in the blend increased the EGT by 31.94% compared to neat biodiesel. Similarly, using n- butanol, the CO formation increased by 33, 29.94, and 69.3% using 10, 20, and 30% n-butanol blend in B100 respectively while compared to neat biodiesel. It is also noted that this may also be due to the increased droplet diameter by the fuel during the injection.

The influence of higher alcohol and vegetable oil in diesel and biodiesel fuelled in CI engine was experimentally studied by Yilmaz and Atmanli^24^. The vegetable oil chosen was soybean oil (10%) and added to the blends of biodiesel (80%) and the remaining 10% of alcohol was added. It was reported that the fuel properties are improved using pentanol and propanol with neat diesel and vegetable oil as a quaternary blend. Vegetable oil exhibited higher kinematic viscosity and lower cetane number and it was reduced by adding alcohol which improved the viscosity of the fuel and exhibited similar cetane properties to diesel. Comparing the performance of diesel, waste oil, and vegetable oil, the fuel with alcohol exhibited better performance compared to neat diesel, and diesel with waste oil and vegetable oil. The improved performance may be due to the higher oxygen content available in the fuel with alcohol content for enhanced combustion. Due to enhanced combustion, the exhaust gas temperature increases which simultaneously increases the formation of NOx. With higher latent heat of vaporization and lower cetane number using higher alcohol content in the fuel leads to the higher formation of CO and HC emissions.

The potential of using vegetable oil, alcohols, blends, and blends of biodiesel on CI engines was experimentally analyzed by Yilmaz and Vigil^25^. The diesel fuel is made constant to 70% with vegetable oil, alcohol, and biodiesel by varying the concentration. It was reported that the blends of biodiesel with vegetable oil, and alcohol increased the HC emission and CO emission during the no load condition whereas, the emissions are reduced at full load condition using ethanol, methanol, and butanol as alcohol additives. It was observed that due to lower exhaust gas temperature the formation of NOx was reduced with alcohol mixed with vegetable oil biodiesel and diesel blend. However, the addition of methanol with biodiesel and diesel blend reduced the formation of NOx while compared to butanol and ethanol blends whereas, the HC emissions were increased as methanol and ethanol exhibited higher heat of vaporization. It was also concluded that to meet the current demand for diesel fuel, the proposed fuel can be an alternative solution.

Atmanli^26^ demonstrated the effects of adding higher alcohols such as n-butanol and 1-pentanol with diesel on the emission of the DI CI engine. The addition of higher alcohols influenced in reduction of the temperature of the combustion resulting in a reduction of NOx and simultaneous reduction in the thermal efficiency of the engine.

In the pursuit to achieve semi- low temperature combustion Atmanli^27^ investigated the performance of the binary blends of diesel and biodiesel obtained from waste cooking oil and C3, C4, and C5. Usage of binary blends shoes the characteristics of S-LTC in diesel engines, benefitted by ultra-low NOx and CO but, traded off by reduced efficiency of the engine and also studied the performance of the Diesel/1 Pentanol blends in two different engines. The results showed promising performance for both the engine configurations without any modifications^28^.

Siti Nurul Akmal Yusof et al.^29^ carried out a complete overview based on the nanoparticle usage in the fuels (individual tradition fuels, biodiesel from vegetable oils, alcoholic fuels, and synthetic fuels) fuelled in IC engines. These compile the details of the engine's outcomes such as performance impact, behavior on combustion, and impact on the exhaust gas emission from the engine while using the fuels with various nanoparticles with different methods and sizes. They obviously point out fuels of engine mixed with various Nanoparticles such as Zirconium nanoparticle, Copper nanoparticle, Nickel nanoparticle, Aluminium oxide nanoparticle, Zing oxide nanoparticle, Cerium oxide nanoparticle, Manganese oxide nanoparticle, silver oxide nanoparticle, Titanium oxide nanoparticle, and Copper oxide nanoparticle. They lead to the desired emission reduction in the fuels of the engine.

Balasubramanian et al.^30^ investigated aluminum oxide nanoparticles (10 ppm, 20 ppm, and 30 ppm) in lemon grass oil biodiesel-based performance in CI engine with direct injection. The superior collaboration of the nanoparticle in lemon grass oil biodiesel was homogeneously mixed very effectively using an ultrasonicator. They concluded that the 20 ppm of aluminium oxide nanoparticle mixing with lemon grass oil biodiesel produced higher thermal efficiency and reduced the emission of the engine exhaust when compared with the other two, and the traditional diesel fuel in the same operating conditions. This may be due to the oxidation properties of the nanoparticles in the biodiesel blend.

Sathiamurthi et al.^31^ used Al_2_O_3_ with different sizes such as 40 to 50 nm and varied the concentration of nanoparticles from 0.5 to 2 g per liter of the fuel prepared for testing. They concluded that the addition of the nanoparticle leads to the diesel fuel with the improvement in output parameters such as performance and combustion. The exhaust emission such as CO, HC, NOx, and smoke are also reduced due to the oxygenating properties when compared with the biodiesel blend without nanoparticles.

Gumus et al.^32^ studied diesel engine performance with oxides of aluminium (50 ppm) and oxides of copper (50 ppm) with diesel. Diesel with nanoparticles of aluminum oxides provides better results on emission when compared with Diesel with copper oxide nanoparticles. The usage of Nanoparticles showed a decrease in fuel consumption from the engine and also helps to reduce the NOx, HC, and CO emissions from the exhaust of the engine^33–35^. Recycling the waste things into useful products is a good approach to creating the required things with availability.

So, every day there is a huge amount of fish waste were available in our country in their corresponding handling places. So, the conversation of this waste into useful biodiesel is very helpful for the future and further improvisation of that biodiesel is also an essential one. This experimental study is mainly focused on focused the comparison of the five test fuels such as neat diesel, neat Biodiesel of Waste Fish Oil (100%), 20% blend of biodiesel of waste fish oil (BDWFO20), Aluminium oxide nanoparticles mixed Biodiesel of Waste Fish Oil (BDWFO100 with AN additive) and Aluminium oxide nanoparticles mixed in 20% blend of biodiesel of waste fish oil (BDWFO20 with AN additive) and it is tested for the same operating conditions. Alumina (Al2O3) nanoparticles have drawn the most attention among the various types of nanomaterials-based additives for biodiesel because of their exceptional chemical and physical properties, excellent ability to disperse in biodiesel for improved performance and reduced emission wit excellent combustion characteristics. The corresponding results related to the CI engine were considered and preferable output-based fuel is recommended as an alternate fuel.

## Experimental procedure

The waste fish oil is produced through the wastes (unwanted parts/portions) of fish from the fish market/fish production industry^14–18^. The produced waste fish oil collected up to 30 L. Then the oil is processed by the acidic and base transesterification process^1^ and^3^ to obtain the biodiesel. Initially, the oil is maintained at 60 °C by constant temperature and maintainable heating for thirty minutes with string. Then the alcohol concentration such as methanol is added up to 250 mL per liter concentration. So nearly 7.5 L of methanol is poured into that container that contains the waste fish oil and it is maintained at a constant temperature with continuous heating. Then this involvement was maintained at a similar temperature means of half an hour.

At that point, an acidic catalyst such as H_2_SO_4_ is used 150 mL is poured into the container which is based on 5 mL per litter proportion of the waste fish oil. Then all this mixture was maintained at the same temperature for up to 60 min. Then the heated oil is placed into the separate inverted conical setup for up to 30 h to settle the glycerin. After that, the settled glycerin was collected and the remaining oil is again heated at the same temperature. This time base catalyst such as KOH is poured into that on the basis of 5 g/L. So, a total of 150 g of KOH is poured and the oil is maintained at the same temperature for up to 60 min.

**Figure 1:**
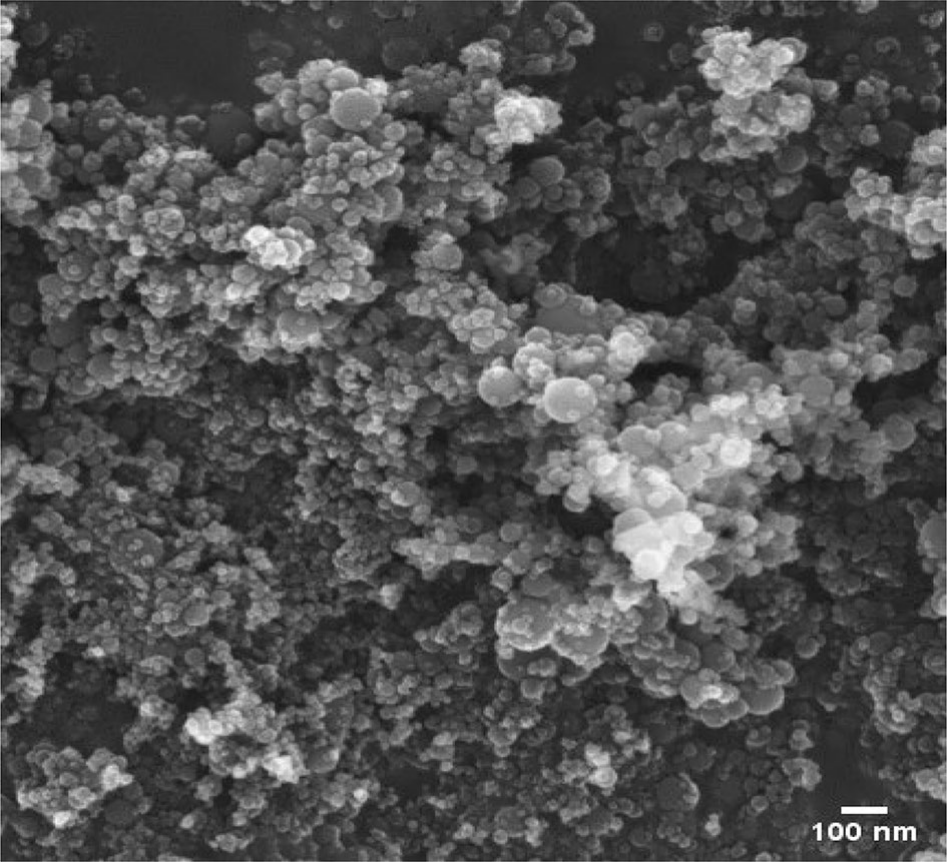
Aluminium oxide nano particles.

After that, the oil is again cooled in the air with that same inverted conical setup and maintained for 30 h. Finally, the biodiesel is separated after removing the glycerin settled down in the arrangement. This biodiesel is cleaned with the water then the cleaned biodiesel is again heated at 45–50 °C for 30 min to remove the moisture content. Finally, 92% yield of biodiesel is produced from these steps. The sample of biodiesel of waste fish oil (BDWFO) is shown in Fig. 1.

There are five combination fuels were considered for this investigation.

1. 100% of individual diesel is named D100.
2. 100% of biodiesel of waste fish oil is named BDWFO 100.
3. 80% of diesel with 20% of BDWFO blending in the volume bass is named BDWFO 20.
4. BDWFO with 200 ppm of aluminium oxide nanoparticles mixed fuel is named as BDWFO with AN.
5. BDWFO 20 with 200 ppm of aluminium oxide nanoparticle mixed fuel is named BDWFO 20 with AN.

The Aluminium oxide nanoparticles used in the present experimental investigation in the average size of 30–40 nm is shown in Fig. 1 based on the literature^20–26^. 200 ppm of Aluminium oxide nanoparticles (AN) is taken^19–25^ and mixed in neat BDWFO and blended with BDWFO20. The nanoparticle is mixed with biodiesel blends with the help of an ultrasonicator have the power of 500 W with a 20 kHz frequency. This equipment is very useful to mix the nanoparticle with the fuel in a clear manner by maintaining the frequency for a certain period of time in this place 45 min at room temperature to obtain the grate mixing with the fuel. The stability behavior of nanofluid is tabulated in Table 1. The zeta potential of BDWFO20 and BDWFO100 was tested with 200 ppm of Al2O3 nanoparticle, and the results were determined to be 118.2 and 116.8 mV, respectively.

**Table 1:** Stability behavior of nanofluids.

Behaviour on stability	Zeta potential range (mV)
Excellent	> ± 60
Good	± 40–± 60
Moderate	± 30–± 40
Incipient	± 10–± 30
Coagulation (rapid)	0–± 5

**Figure 2:**
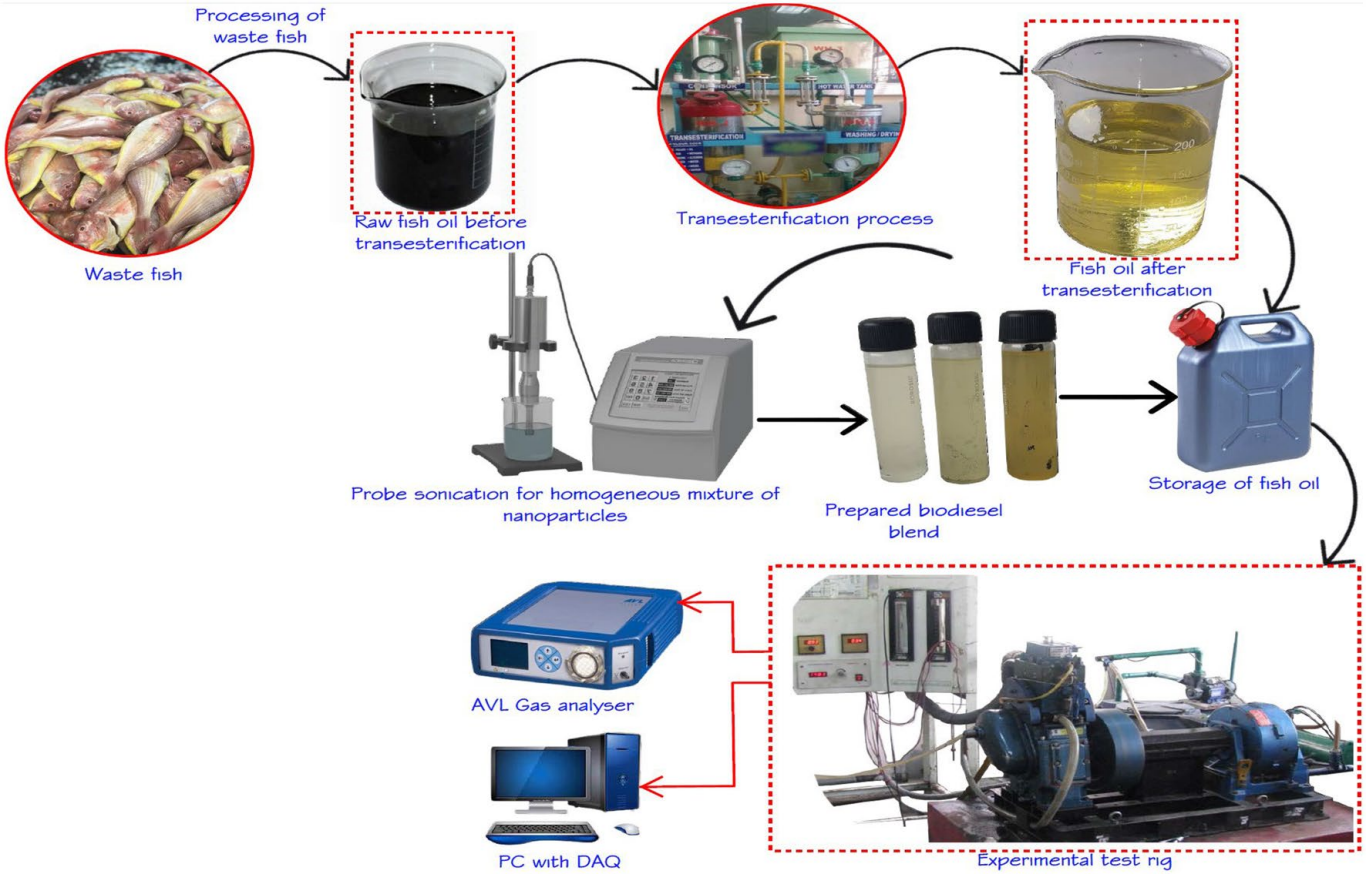
Schematic diagram on the production of waste fish oil through transesterification process and its application in compression ignition engine.

The above mentioned combination of fuels is working in the following engine. Single-cylinder naturally aspirated compressed ignition engine with 5.2 kW power at the maximum speed of 1500 rpm (Kirloskar engine). Eddy current loading is used. There is an airflow sensor, fuel flow sensor, pressure transducer, and the dynamic encoder in the dynamometer arrangements is placed in the corresponding position which is clearly shown in Fig. 2. All those sensors and transducers were clearly connected with the Data acquisition system which is connected with the computer system. This system leads to measuring the performance and emission-related readings with respect to the percentage of loading. The details of engine with specification is tabulated in Table 2 and their uncertainty is tabulated in Table 3.

**Table 2:** Test rig specification.

Engine make	Kirloskar TV1 diesel engine
Bore and stroke	87.5 mm and 110 mm
Piston type	Hemispherical bowl
Number of cylinder	1
Type of engine cooling	Water cooling
Rated power	5.2 kW
Rated speed	1500 rpm
Loading type	Eddy current dynamometer
Compression ratio	17.5:1
Fuel injection	21 obTDC
Injection pressure	210 bar
Fuel tank capacity	6 L

**Table 3:** Uncertainty of measuring instrument.

Parameter	Uncertainty (%)
Speed	± 1.5
BTE	± 0.7
BSFC	± 0.8
Pressure	± 0.6
Crank angle	± 1.1

Exhaust emission gas related parameters are measured by the smoke meter and AVL gas analyzer. The specification of the AVL Digas 444 analyser is tabulated in Table 4. The range of the measuring instrument and their uncertainty are listed in Table 5. The test fuel is stored in the secondary fuel tank. The corresponding flow is passed into the engine there are four loading condition related results were taken for the investigation such as ¼ load, ½ load, ¾^th^ load, and a full load of the engine capacity. Every reading was taken after running the engine under the same conditions for up to 20 min to obtain an accurate reading with precession. Similarly, corresponding exhaust emission output is measured by the AVL gas analyzer for the gases like Carbon monoxide, Carbon Dioxide (CO2), Unburnt Hydrocarbon (UHC), and Nitrogen oxides (NOx). The same procedure is followed for all the fuels considered in the experimental arrangement.

**Table 4:** Specifcation of gas analyser.

	Make	AVL digas 444
AVL gas analyser	Time for warm up	7 min
	Relative humidity	95%
	Response time	15 s

**Table 5:** Range of measuring parameters of emission gas analyser.

Parameter	Range	Uncertainty (%)
CO	0–15% by vol	± 0.3
UHC	0–3000 ppm	± 0.4
NOx	0–5000 ppm	± 0.5
CO2	0–25% by vol	± 0.3

## Results and discussion

The fuel properties of biodiesel blends and conventional diesel measured using ASTM testing standards are tabulated in Table 6. From Table 6, it is clear that the density of fuel increases with the increasing composition of biodiesel and biodiesel with the nanoparticle. Among that BDWFO 20 with AN have a higher Calorific value and Cetane Number. BDWFO 100 with AN has a higher flash point and kinematic viscosity.

**Table 6:** Properties of diesel, biodiesel, and biodiesel with nanoparticles.

Properties	Fuels				
	D100	BDWFO 100	BDWFO 20	BDWFO 100 with AN	BDWFO 20 with AN
Density in kg/m^3^	827	844	839	881	851
Calorific value in kJ/kg	45,480	40,700	42,900	45,584	45,590
Cetane number	52	48	51	54	56
Kinematic viscosity in cSt @ 40 °C	3.05	3.5	3.17	3.9	3.6
Flash point in °C	52	84	73	94	82

### Performance characteristics

**Figure 3:**
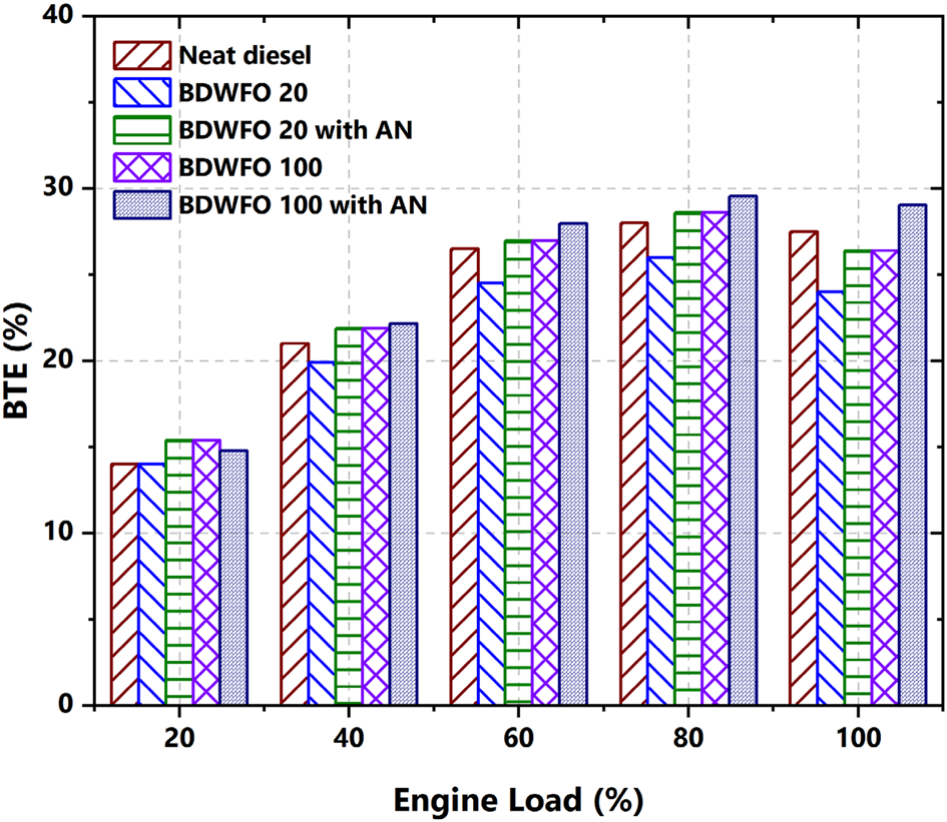
BTE—brake thermal efficiency (%) with respect to Load variation (%).

The variation of brake thermal efficiency for different engine loads of prepared biodiesel blend and neat diesel is calculated and plotted in Fig. 3. From Fig. 3 it is observed that the BDWFO20 and BDWFO20 with nanoadditive exhibit lower thermal efficiency of about 13 and 3.87% respectively compared to neat diesel at higher engine load, while the BTE of the engine for the same operating condition is improved using neat BDWFO with higher loading of nanoadditive exhibited higher BTE of about 6% than neat diesel fuel. Similarly, the neat BDWFO exhibited lower thermal efficiency of about 4% than diesel fuel. The blended and individual biodiesel efficiency is increased with the help of the Aluminium oxide Nanoparticles as the surface to volume ratio of the fuel increases. The increase in the BTE may be due to the greater oxidation created by the Nanoparticle in the fuel during the combustion process as well as the higher cetane number and calorific values of that particular fuel leading to the increase in the efficiency in the considered engine.

**Figure 4:**
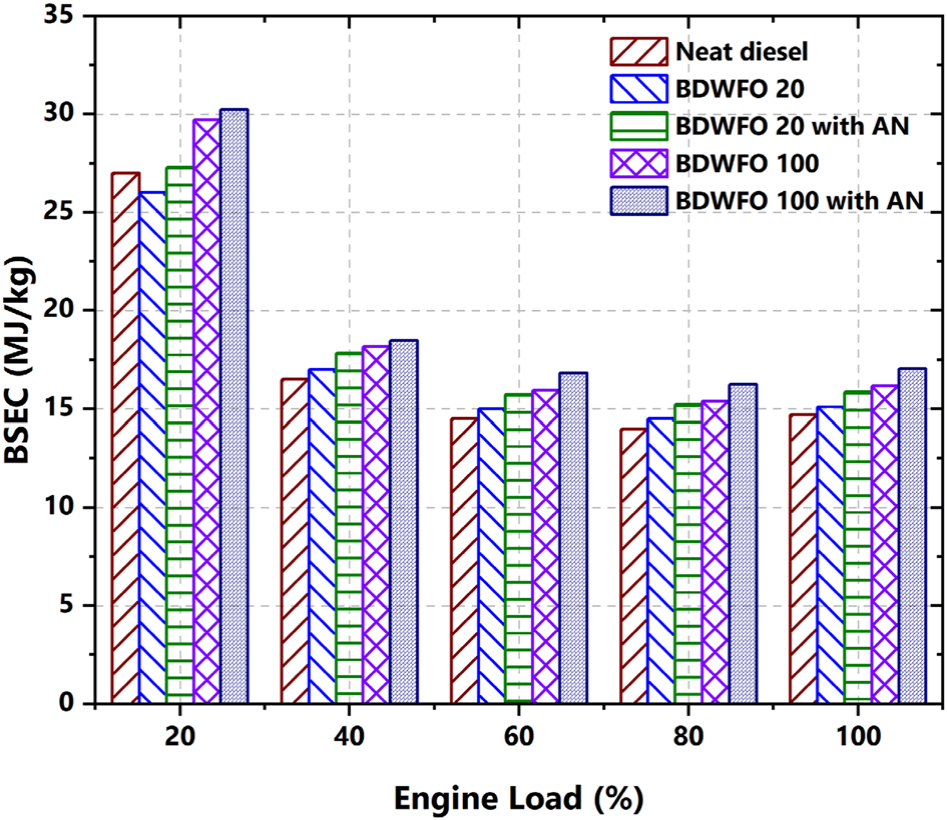
Brake specific energy consumption—BSEC (MJ/kW h) on various loads of engine (%).

The performance parameter based on the variation of the load the brake-specific energy consumption (BSEC) is plotted in Fig. 4. From the results, it is found that the neat biodiesel and biodiesel BDWFO20 with and without nanoadditive exhibit higher thermal efficiency compared to neat diesel fuel. There is an improvement of about 10, 3.6, 16, and 8% in the BSEC from the engine fuelled using BDWFO100, BDWFO20, BDWFO20 with AN, and BDWFO20 with AN respectively as an additive in the biodiesel blend compared to neat diesel fuel. The BDWFO100 and BDWFO100 with AN have improved the BSEC of the engine as the fuel exhibits higher oxygen content. Individual biodiesel consumption is more to produce the same energy. These deviations are due to the greater Cetane number and calorific values in all the fuel combinations. Also, the greater combustion duration leads to increased efficiencies in the fuel involved in the internal combustion engine.

### Combustion characteristics

The combustion characteristics of any engine can be analyzed by critical examination of pressure and heat release trends for individual crank angles at full load operation of the engine.

#### In-cylinder pressure variation

**Figure 5:**
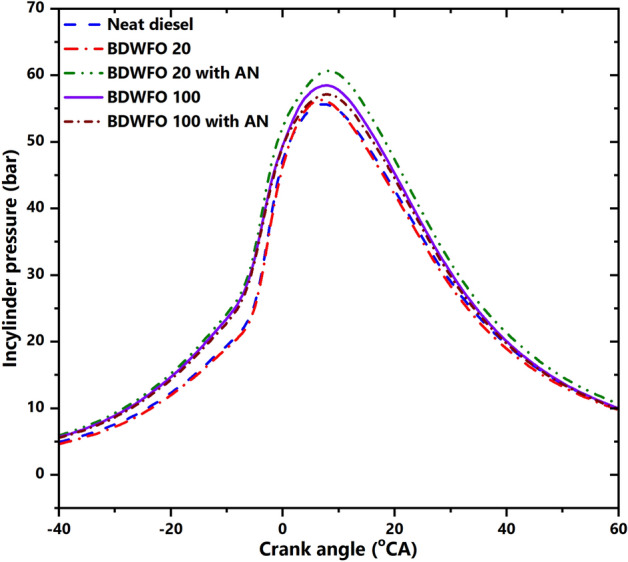
In cylinder pressure (bar) variations related to the crank angle (CA).

Figure 5 shows the plot between in-cylinder pressure and the crank angle at full load operation of the engine. From the curve, it is evident that the peak pressure for all the fuel blends falls between 10 and 22 degrees of crank angle. From the data obtained we can see that the peak pressure of the blends is higher than that of the diesel irrespective of the composition of the fuel. This increasing trend in pressure can be seen as the consequence of the higher calorific value of the fuel with biodiesel and nanoparticle as additives. The increase in the in-cylinder pressure from the engine fuelled using BDWFO 20 with AN, BDWFO 100 with AN, BDWFO 100, and BDWFO 20, when compared with diesel fuel are found as 16%, 14.9%, 9.5%, and 9.5% respectively. The cetane number increase characterizes the better auto ignition property of the fuel added to it and the oxygen availability by aluminum oxide nanoparticles plays a vital role in increasing the combustion process and producing high cylinder pressure.

#### Heat release analysis

**Figure 6:**
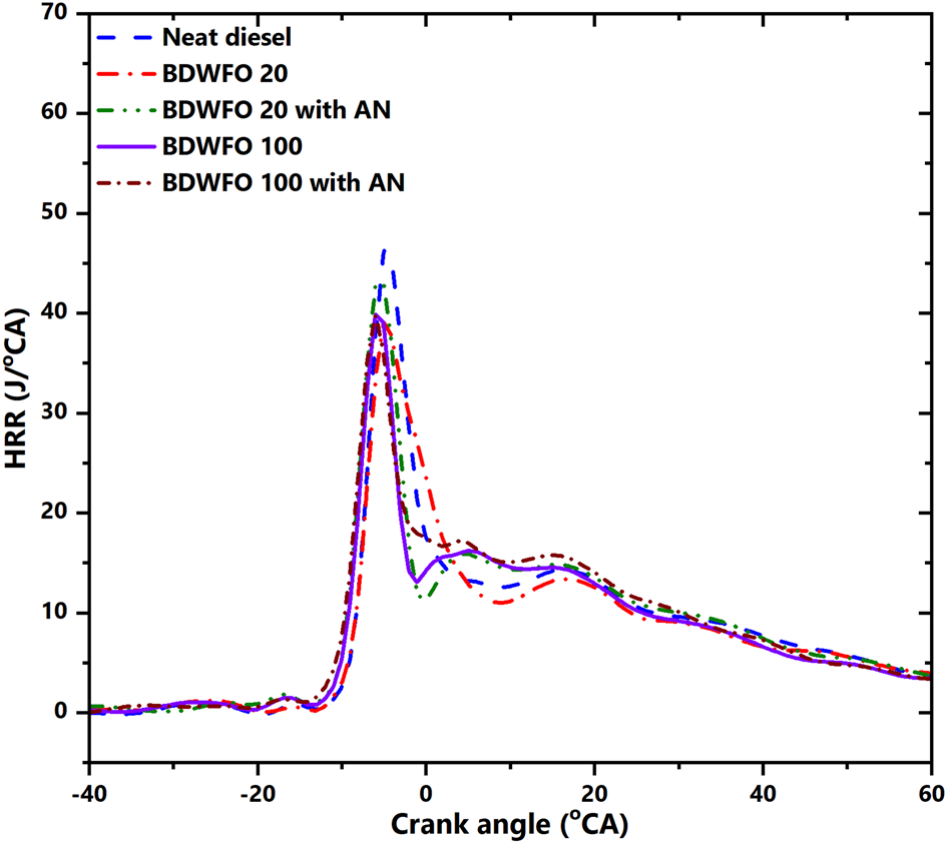
Heat release rate—HRR variations related to the crank angle (CA).

The heat release analysis can be done by analyzing cumulative heat release and heat release rate of the combustion. Figure 6 shows the variations in the rate of heat release with respect to crank angle for full load operation of the engine. From the figure, it is easily seen that the heat release rate is minimum for BDWFO 20 with AN and Diesel showing a higher heat release than all the blends. Even though the blends of Biodiesel with AN have a higher calorific value than the neat diesel it has low heat release, this is due to the high density of the blends. The rate of atomization is a function of fuel density- denser fuel will be poorly atomized. Hence the physical delay of the fuel is increased the combustion gets narrowed in the premixed combustion phase and has a prolonged diffusion combustion phase. Increased diffusion combustion phase will reduce the useful heat release.

**Figure 7:**
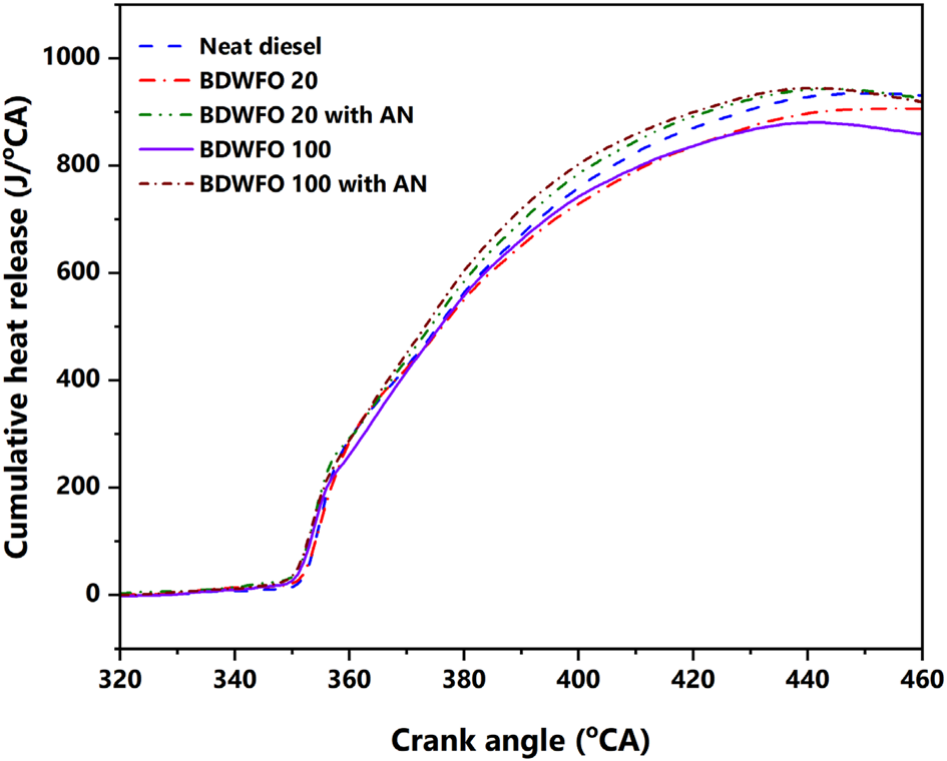
Cumulative heat release with respect to crank angle.

Figure 7 shows the cumulative heat release of the blends at full load engine operation, from the figure it can be seen that BDWFO 20 and BDWFO 100 have reduced heat release than the diesel fuel, and the rest of the blends that have Aluminum oxide nanoparticle has higher cumulative heat release than diesel. Even though the diesel fuel showed a higher heat release rate than biodiesel the cumulative heat released has been reduced. The density of the biodiesel has played an important role in achieving a prolonged diffusion combustion phase and simultaneously increasing the cumulative heat release of the blends when compared with diesel.

#### Ignition delay and combustion duration analysis

**Figure 8:**
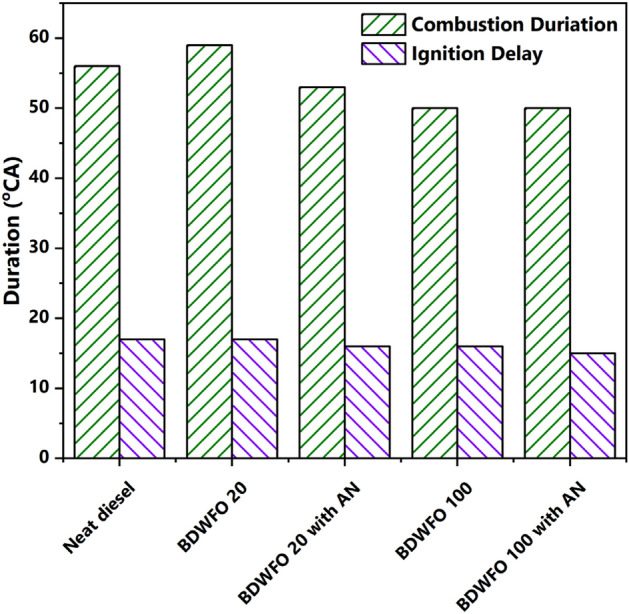
Variation of combustion duration and ignition delay for various fuel blends.

The variation in the combustion duration and ignition delay for the various fuel blends has been given in Fig. 8. From the figure, it is evident that the ignition delay and combustion duration are almost similar for the blends at full load irrespective of their composition. Despite the fact that the density of the blends is higher as the biodiesel and AN increase it has a similar ignition delay. This phenomenon of similar ID can be explained in terms of the increased cetane number of the blends. As the density of the blends affects the atomization of the blends that accounts for an increased physical delay period, on the other hand, an increase in cetane number reduces the chemical delay. The combustion duration for the blends with nanoparticles as an additive is less when compared with the blends without nanoparticles since the extra oxygen given by the blends attempt to increase the combustion rate.

### Emission characteristics

#### CO emission

**Figure 9:**
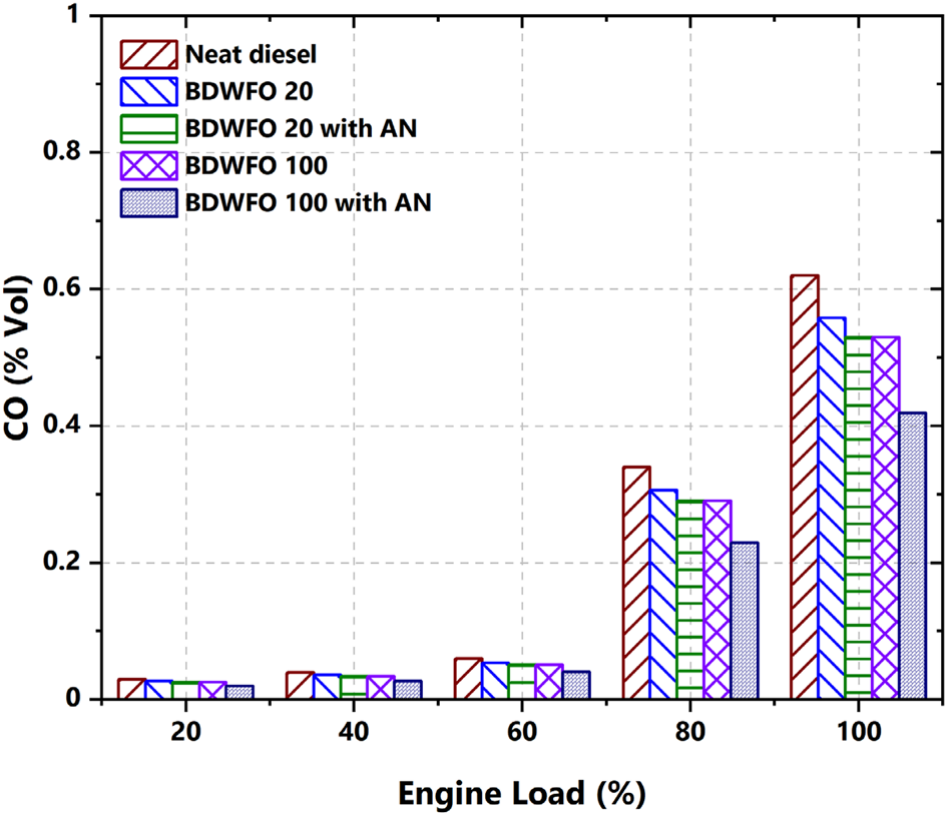
Emission of carbon monoxides versus variation on load (%).

The exhaust gas emissions were measured by an AVL gas analyzer. A substantial source of CO2 emissions from diesel engines plays a major contributor to the formation of ozone. The Carbon monoxide emissions from the exhaust gases in terms of percentage are related to the load variations in Fig. 9. The CO emissions increased with increasing the load. Under full load condition, BDWFO 100 fuel has 16% and BDWFO 20 fuel 5% lesser CO emission from the exhaust gas when compared with the Diesel fuel. Similarly, BDWFO 20 with AN fuel has 5% and BDWFO 100 with AN fuel has produced 9% lesser CO emission in the exhaust gas when compared with the diesel fuel. The maximum and minimum CO emissions were reached by BDWFO 100 and BDWFO 100 with AN respectively. The maximum CO emission emitted from the engine might be due to the lack of oxygenation but Aluminium oxide Nanoparticles lead to the maximum oxidation in the fuel in combustion so it leads to the minimum CO emission.

#### UHC emission

**Figure 10:**
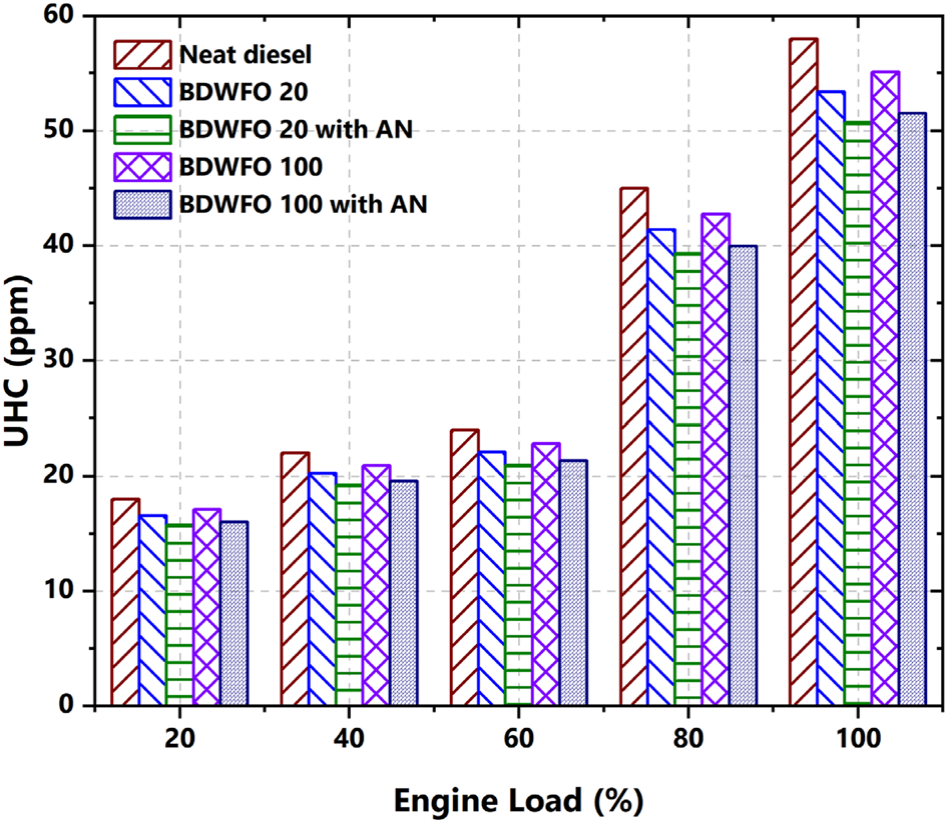
Emission of unburnt hydro carbons—UHC versus variation on load (%).

The unburnt hydrocarbon emission from the exhaust emission with respect to the percentage of load variation is given in Fig. 10. It is augmented with respect to load increment. The BDWFO 100fuels have a 6% greater HC emission when compared with diesel fuel. Correspondingly BDWFO 100 with AN fuel have 1%, BDWFO 20 fuel have 10% and BDWFO 20 with AN fuel have 15% CO emission lesser than the Diesel fuel. The lowest HC emission is achieved by BDWFO 20 with AN fuel. This HC emission is due to maximum incomplete combustion. Blending and Aluminium Oxide Nanoparticle combination leads to the maximum oxidation in combustion which leads to the complete combustion.

#### CO_2_ emission

**Figure 11:**
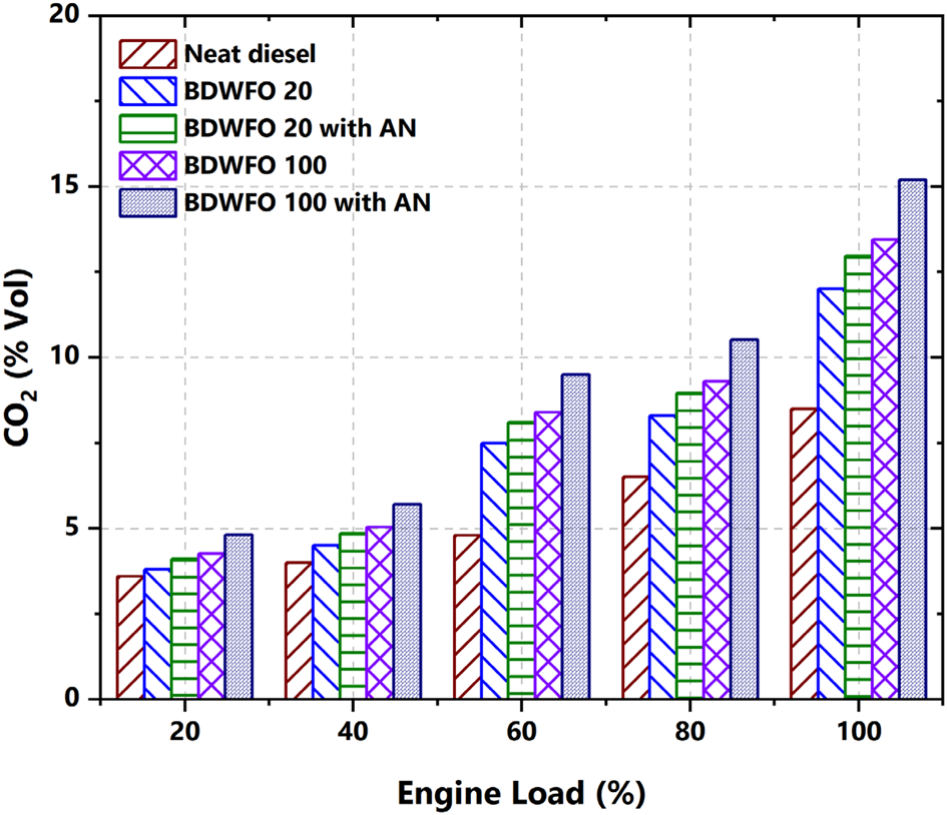
Emission of carbon dioxide—CO_2_ (%) versus variation on load (%).

Carbon Dioxide emission from the exhaust emission with respect to the percentage of load variation is plotted in Fig. 11. When compared with the diesel fuel CO emission with other fuels such as BDWFO 20, BDWFO 20 with AN, BDWFO 100, and BDWFO 100 with AN increased the CO2 emission by 28%, 38%, 43%, and 62% respectively in the exhaust gas with the same engine in same operating condition. Diesel fuels have produced very less CO_2_ emissions whereas, the neat biodiesel with nanoparticles (BDWFO 100 with AN) produced the maximum CO_2_ emission. This higher emission is due to some of the HC emissions and CO emissions being oxidized and which will produce the greater CO_2_. The biodiesel and its blend with Aluminium oxide Nanoparticles lead to the maximum possible oxidation in the combustion process.

#### Formation of NOx

**Figure 12:**
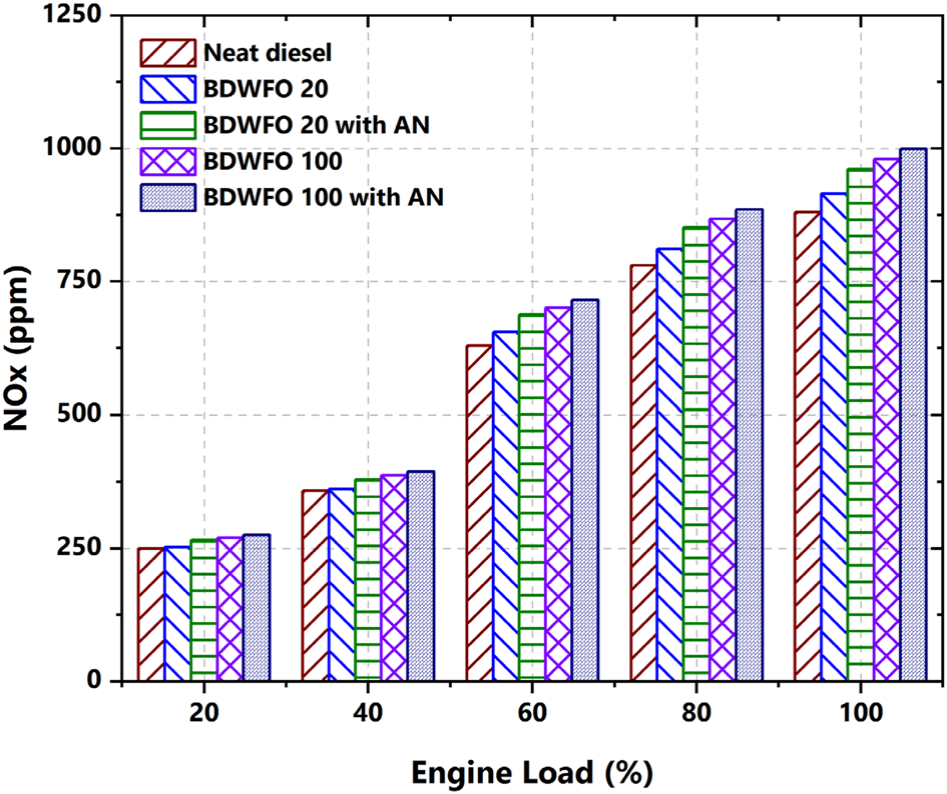
NOx emission—NOx versus variation on load (%).

The formation of NOx is majorly influenced by the availability of oxygen content, time for reaction, and in-cylinder temperature. Figure 12 is plotted for the experimental output results of the Nitrogen oxides in the exhaust emission in the considered engine. The biodiesel blend BDWFO 20 fuel has 4% and BDWFO 100 fuel has 8% higher nitrogen oxides emission in the exhaust gas when compared with the diesel fuel. In the same way, BDWFO 100 with AN fuel has 20% and BDWFO 20 with AN fuel has lesser NOx values when compared to diesel fuel. The highest and smallest NOx emission was obtained by BDWFO 100 fuel with AN and BDWFO 20 with AN in the fuel. This might be due to the higher rate of combustion exhibited by the nanoparticle in the fuel. Additionally, the excess oxygen content in the fuel increase the combustion temperature for higher NOx formation. The higher NOx formation is also due to the increased cetane number of biodiesel. Furthermore, when additional fuel is injected, a rich core is produced, particularly at high loads which may be a reason for higher NOx formation. Because the oxygen atom in biodiesel may be utilized to thermally produce NOx, the presence of this high temperature fuel-rich core is expected to affect NOx emission levels. These values of NOx emission are nearly closer to neat diesel fuel. These all are due to the cylinder temperature and the proper combustion of the cylinder.

## Conclusions

In the present experimental investigation, the effect of higher concentration aluminium oxide nanoparticle on biodiesel derived from waste fish oil on CI engine is analysed for its performance, emission and combustion characteristics and the following conclusions are arrived.

- Based on the performance
  - Efficiencies of other fuels considered were near to the diesel fuel especially BDWFO 100 with AN fuel have 6% higher BTE and BDWFO 20 with AN as additive produced a lower BTE of 4% than diesel fuel.
  - BSEC of all the considered fuels with and without nanoadditives is slightly higher than diesel fuel.
- Based on combustion studies considered fuels were higher in-cylinder pressure and higher heat release rate compared with diesel fuel.
- Based on emission
  - Emission reduction and increase were obtained nearly to the diesel.
  - BDWFO 20 with AN fuel has produced a lower CO, and HC while the CO_2_ emission are higher.
  - Aluminium oxide particles used fuel BDWFO 100 with AN and BDWFO 20 with AN were produced less emission except CO_2_.
- The aluminium oxide mixed waste fish oil produced higher performance and combustion as well as reduced emission when compared with diesel fuel.

## Data Availability

The datasets used and/or analysed during the current study available from the corresponding author on reasonable request.

## Abbreviations

Al_2_O_3_: Aluminium oxide
AN: Aluminium oxide nanoparticle
BDWFO 100: 100% BDWFO
BDWFO 20: 20% BDWFO + 80% diesel
BDWFO: Bio diesel of waste fish oil
BSFC: Brake specific fuel consumption
BTE: Break thermal efficiency
CO: Carbon monoxide
CO_2_: Carbon dioxide
D100: 100% of diesel
DI: Direct injection
EGT: Exhaust gas temperature
HC: Hydrocarbon
HRR: Heat release rate
ICP: In-cylinder pressure
NOx: Nitrogen oxides
P: Pressure (bar)
Ppm: Parts per million
θ: Angle (°)
